# Omnichannel and Product Quality Attributes in Food E-Retail: A Choice Experiment on Consumer Purchases of Australian Beef in China

**DOI:** 10.3390/foods14101813

**Published:** 2025-05-20

**Authors:** Yaochen Hou, Shoufeng Cao, Kim Bryceson, Phillip Currey, Asif Yaseen

**Affiliations:** 1 Arafura Strategic Advisory Ltd., Sheung Wan 00852, Hong Kong; 2 School of Agriculture and Food Sustainability (AGFS), The University of Queensland, Brisbane, QLD 4072, Australia; s.cao@uq.edu.au (S.C.); phil@philcurrey.com.au (P.C.); yaseen.asif@gmail.com (A.Y.); 3 Department of Commerce, Bahauddin Zakariya University, Multan 60000, Pakistan

**Keywords:** e-retail, omnichannel retailing, Australian beef, product attribute, consumer perception, purchase behaviour, willingness to pay, discrete choice experiment

## Abstract

With the rise of omnichannel (OC) retailing in food e-retail, understanding how OC retailing and product quality attributes influence consumer purchasing behaviour and value perceptions is crucial for developing e-retail strategies and enhancing consumer services. This study examined their impacts on Chinese consumers’ purchases of Australian beef (brisket) through a discrete choice experiment in Beijing, Shanghai, Guangzhou and Shenzhen and analysed 872 valid responses using multinomial logit, random parameter logit, and latent class models. Our findings reveal that Chinese consumers prefer buying Australian brisket via OC apps and offline stores, paying approx. 44% and 134% more per 500 g, respectively, compared to self-operated e-commerce stores. Brand, manufacturer and origin traceability are key quality attributes, with additional paid for brisket manufactured and packaged in Australia (under Australian brands) and featuring the MLA “True Aussie Beef” label over QR codes. This study also identified four distinct consumer clusters: (i) premium shoppers, (ii) channel and traceability-oriented shoppers, (iii) omnichannel and price-oriented shoppers and (iv) tech-savvy and discerning shoppers, highlighting varying sensitivities to e-retail channels and product attributes. These findings offer strategic and actionable insights for Australian beef exporters and OC retailers seeking to optimise consumer engagement and value creation in China’s evolving e-retail landscape.

## 1. Introduction

China’s imported beef market is the fastest-growing globally, driven by strong beef consumption [1]. China has exponentially imported Australia’s beef products since around 2012 and has become Australia’s top beef export destination in 2019 [2]. Food service and retailing segments dominate China’s import beef market [3]. Amid the intense competition and price-driven nature of the food service channel, the retail segment appeals to consumers who are open to new experiences and attentive to branding [3]. The sale of beef products through China’s e-commerce platforms is well established, providing a modern outlet for delivering premium protein into the diets of Chinese consumers [4]. Omnichannel (OC) retailing integrates online and offline channels to cater to diverse consumer needs and provide the best possible shopping experience across all channels [5,6]. OC represents a recent and significant revolution in contemporary e-retail strategy and embraces various channels and product attributes to engage with diverse consumers for order fulfilment [6]. It thereby contributes more than the existing consumer experience in terms of product demonstration, brand-consumer engagement and sales growth [5]. Studies, such as Wang et al. [7], have revealed that food choices among consumers vary significantly among various e-retail models. Therefore, it is valuable to understand Chinese consumers’ preference for OC retailing in the purchase of Australia’s beef products within the e-retail environment.

Chinese consumers have rapidly adapted to purchasing groceries through OC retailing, driven by the dynamic shifts in grocery shopping behaviours and the widespread acceptance and adoption of OC retailing in China [8]. The work by Akter et al. [9] has identified that customer engagement is an OC management capability in international marketing. As consumers’ evaluation of products is determined by such extrinsic characteristics as price, brand information, manufacturer name and country of origin [10], engaging with consumers to understand their perceptions and effectively communicating these product quality attributes is highly important in the OC environment. Brands are signals of product quality by consumers [11,12] and play an important role in consumer buying decisions [13]. Country of origin is also a highly valued product quality attribute [14] as it not only serves as a gateway of the distribution channel [15] but also steers consumer perception of product quality [16]. Recent studies have revealed country of origin’s interactions with pricing strategies [17] and brand reputation [18]. Traceability is another important product quality attribute [19] as it can deliver additional consumer value [20]. It is, therefore, valuable to examine Chinese consumers’ perception of these quality attributes associated with Australian beef products within the e-retail landscape.

This study explores a gap in the literature around how shopping channel selection interacts with product quality attributes in influencing consumer purchases of Australian beef within China’s e-retail landscape. China is a growing beef market and Australia’s largest beef export market [2], highlighting the need for tailored marketing strategies to engage consumers through e-retail channels. The aim of this study is to understand consumer preferences for OC retailing and product quality attributes, providing insights into how to integrate OC retailing with product quality attribute communication to optimise product offerings, service delivery, and value creation [21]. Through a discrete choice experiment (a survey-based experimental method), this study investigated consumer purchases of Australia’s beef (brisket) through e-retail channels in China’s four metropolitan cities: Beijing, Shanghai, Guangzhou and Shenzhen. The novelty of this study lies in examining how OC retailing and various product quality attributes, including brand and manufacturer location, as well as country-of-origin labels, influence Chinese consumers’ purchases and willingness to pay (WTP) for Australia’s beef and highlight their integration in shaping e-retail strategies and improving consumer services.

This study makes significant contributions to international food e-retail from a dual perspective. Theoretically, this study builds upon the literature on OC consumer engagement [9] to contribute to the signalling theory [11] by demonstrating how product quality attributes, including the brand, manufacturer location and traceability signals, shape consumer trust and WTP in food e-retail. Additionally, by identifying four consumer clusters based on channel and product quality attribute preferences, this study advances segmentation research in international food marketing in OC environments. Practically, this study provides actionable insights for Australian beef exporters and OC retailers by highlighting the importance of integrating product quality attribute communication across multiple e-retail channels to enhance consumer purchases and WTP. These insights are essential for exporters navigating China’s evolving e-retail market for value creation.

The remainder of this paper is structured as follows: Section 2 reviews extant work related to OC retailing and product quality attribute signals. Section 3 presents a discrete choice experiment approach employed in this study. This is followed by the main results (Section 4), discussion, limitations of this study and future research avenues (Section 5). Section 6 concludes this paper with key findings and valuable insights.

## 2. Literature Review

### 2.1. Omnichannel Retailing and Consumer Choice Behaviours

Omnichannel (OC) retailing represents an evolution of multi-channel retailing, which focuses on combining physical stores with phone, web and mobile channels to maximise retail performance [22,23,24]. While OC retailing follows the multi-channel strategy to interact with consumers through various online and offline channels and touchpoints, it adopts a consumer-focused strategy to deliver a unified shopping experience by allowing consumers to switch freely across all channels [5,6,23]. The rise of OC retailing is fuelled by the penetration of the Internet, mobile technologies and digital technologies into the retail landscape and increasing digital experience, as well as dynamically changing browsing and shopping behaviours [25]. Due to the seamless shopping experiences across different channels, consumers have shown a strong preference for OC retailing. This is evidenced by a survey of over 46,000 retail customers in the U.S. from June 2015 to August 2016. This survey showed that 73% of the participants were OC consumers compared to 20% store-only shoppers and only 7% online-only shoppers [26]. This trend is also evidenced in China, where more than 70% of consumers actively engage in omnichannel shopping [27]. The transition from e-commerce to OC retailing [28] and the boom of OC retailing in China [29] emphasise the strategic importance of OC retailing. This shift acknowledges the changing retail landscape and the increasing consumer expectation for an integrated shopping experience across multiple channels.

OC retailing has attracted increasing interest among academics and practitioners [23,30]. Research has shown that OC consumers buy more often and spend more compared to store-only shoppers and online-only shoppers [26]. This trend is echoed by a recent study from Symphony Retail AI, which reveals that OC grocery shoppers shop more frequently and spend up to 20% more than their in-store-only counterparts [31]. The unique characteristic of OC shopping is that it allows consumers to have access to different channels in their shopping journey [32]. It is, therefore, crucial to understand consumer perception and preference towards OC retailing with increased flexibility and options offered [23]. Chopra [5] identified product and customer characteristics in channel selection and proposed an OC portfolio to tailor the specific fulfilment of customer requests. Another study by Xu and Jackson [21] examined customer channel selection intention in the OC retailing environment by conducting surveys of U.S. and UK customers. They found that customer behaviours in channel selection are positively impacted by channel transparency, channel convenience, and channel uniformity. A recent systematic literature review conducted by Wolf and Steul-Fischer [30] examined the factors influencing customers’ OC choices. They revealed that customers’ OC choices are influenced by both direct factors, such as perceived channel characteristics and customer needs, and indirect factors, such as customer characteristics and product/service characteristics. These studies have identified the channel choice issues in the OC retailing environment. However, it remains unclear whether consumers have specific channel preferences among various channels and whether consumers’ WTP varies across these channels in China. In response, this study examines consumers’ purchase preferences and WTP for food products—specifically Australia’s beef—within China’s e-retail environment.

### 2.2. Omnichannel Strategies and Product Quality Attributes

In an OC retailing model, customers and retailers engage in three parallel flows of information, product and money as in other supply chains [5]. An OC retailer can market information and products to consumers through a variety of channels, including face-to-face in a retail store or remotely in online shopping [33]. Previous research has revealed that a retailer’s communication strategy significantly influences consumers’ purchase channel choices [34] and purchasing decisions [35]. The prevalence of food fraud accidents has highlighted that consumers have a higher desire for effective communication of product quality attributes [36] and supply chain transparency (traceability) [21] within the retail setting. Therefore, incorporating food attributes in OC communication, such as brand and manufacturer location and country-of-origin traceability, is an important factor in improving customer satisfaction.

Chinese consumers have shown a strong interest in product provenance when shopping for food products due to frequent food fraud incidents [37]. This highlights the importance of effectively communicating food traceability to Chinese consumers [38]. The Australian beef industry has already launched traceability communication programs that communicate beef traceability either by attaching the “True Aussie Beef” label to the retail package or using a Quick Response (QR) code for product history queries to convince Chinese consumers of the provenance and authenticity of the beef products they buy and consume [36]. Despite the adoption of these communication programs by many OC retailers, there is still limited understanding of their effectiveness in convincing consumers and whether there are variations in WTP based on different product communication approaches. This paper attempts to offer a better understanding of the value added through different communication programs and offer insights into adopting alternative communication strategies in OC retailing to enhance customer service.

## 3. Methodology

### 3.1. Discrete Choice Experiment

This study utilised a discrete choice experiment (DCE), implemented as a survey-based experimental method, to examine consumers’ purchase preferences and willingness to pay for OC and product quality attributes. The DCE used a 500 g Australian brisket as a hypothetical product, focusing on key choice attributes. The selection of brisket among diverse beef cuts for the DCE was based on the Chinese consumer demand specification. China imports a variety of beef cuts from international markets, with brisket being the most popular cut, accounting for 22% of Australian beef exports to China [3]. As validated by sales managers from China’s e-commerce and OC companies, brisket is among the best-selling items in the Australian beef category on e-commerce platforms.

This DCE draws on Lancastrian consumer theory [39] and random utility theory [40], which suggest that consumers gain utility from choice attributes and select options that maximise their utility. The random utility (*U_ni_*) for consumer n choosing alternative i is expressed as:

*U*_*ni*_ = *V*_*ni*_ + 𝜀_*ni*_ (1)

where *V_ni_* is the deterministic utility from observable attributes, and $\varepsilon_{ni}\;$ is the stochastic error term. Assuming a linear evaluation of product attributes, *V_ni_* can be expressed as:

(2) $$V_{ni}=\sum_{k=1}^{K}\beta_{nik}V_{nik}$$

where $V_{nik}$ represents the utility that consumer n derives from the k-th attribute of option i, and *β*_*nik*_ is the parameter that reflects the significance of that attribute. The overall random utility of option i for consumer n is expressed as:

(3) $$U_{ni}=\sum_{k=1}^{K}\beta_{nik}V_{nik}+\varepsilon_{ni}$$

j denotes any other options in the choice pool. When *U_ni_* > *U_nj_*, option i is preferable over option j for consumer n. When selecting the alternative offering of the highest utility, the probability P of consumer n opting for option i from a pool of J choices can be described as:

(4) $$P_{ni}=P\left(U_{ni}>U_{nj},\;\forall j\neq i\in J\right)=P\left(V_{ni}+\varepsilon_{ni}>V_{nj}+\varepsilon_{nj},\;\forall j\neq i\in J\right)\;=P\left(\varepsilon_{nj}<\varepsilon_{ni}+V_{ni}-V_{nj},\;\forall j\neq i\in J\right)$$

#### 3.1.1. Identification of Attributes and Corresponding Levels

The selection of choice attributes and their corresponding levels followed a three-stage process to ensure both theoretical grounding and practical relevance. In the first stage, existing literature was reviewed to identify four high-level attributes, including shopping channel [34], brand and manufacturer location [41], country-of-origin traceability [19] and price [10]. This was followed by assigning the number of levels to each attribute with consideration of the need to minimise the cognitive burden on respondents while maintaining sufficient variation for meaningful trade-off analysis. Attributes with broader variability in real-world markets, such as shopping channels and price, were assigned four levels to capture a wider range of consumer choices and purchasing conditions. Brand/manufacturer location and country-of-origin traceability were each represented with three levels, reflecting the typical range of differentiation perceived by Chinese consumers. The configuration—four levels for shopping channels, three for brand/manufacturer location, three for country-of-origin traceability and four for price—was further confirmed based on consumer input and practical realism.

In the second stage, three consumer focus groups were convened to redetermine the number of levels and identify specific attribute levels for each high-level attribute. Each group comprised 8–12 participants, who were selected based on purposive sampling to ensure diversity in region, age, gender and purchasing habits. Facilitated by the first author of this paper, these consumer focus group discussions were conducted via online meetings using WeChat, which is an instant messaging App used widely in China. The consumer-focused stage helped to identify key specific attributes from the consumer perspective and confirmed the relevance of the theoretically selected high-level attributes in a real-world setting.

In the third stage, consultations with eight industry experts from six leading e-commerce and new retail enterprises in China were conducted to validate the practical importance of the attributes and levels. These experts held senior roles in their respective companies and had at least three years of experience dealing with beef sales and marketing through e-commerce and new retail channels. Their input was used to assess and validate the practical importance and feasibility of the selected attributes and levels. By integrating insights from both consumers and industry professionals, the final attribute selection achieved a balance between consumer preferences and industry applicability. Table 1 presents the final choice attributes and respective attribute levels.

The “shopping channel” attribute encompassed four levels: (i) self-operated e-commerce stores; (ii) third-party stores on the e-commerce platforms; (iii) OC retail phone apps; and (iv) OC retail offline grocery stores. The first two levels are traditional e-commerce channels, while the last two are new OC channels.

As consumers cannot physically interact with products in e-retail environments and rely entirely on external information to make purchasing decisions, this study focused on the available extrinsic product cues, including brand and manufacturer location [41], country-of-origin traceability labels [19] and price [10]. The “brand and manufacturer location” attribute for Australian-sourced beef has three levels: (i) Australian brand, manufactured in Australia; (ii) Australian brand, manufactured in China; and (iii) Chinese brand, manufactured in China.

The “country-of-origin traceability” attribute includes (i) QR code—one of the digital tools facilitating information access to deliver traceability information to consumers; (ii) MLA “True Aussie Beef” label—the commonly used medium to promote the origin of Australian beef products in export markets; and (iii) None—indicating no traceability data.

The retail price attribute has four levels, ranging from CNY 35 to 80 for 500 g of Australian brisket. These price levels were set based on the retail prices of equivalent quantities of the product sold by China’s largest e-commerce businesses (e.g., JD.com, Taobao, T-mall and Suning) and OC retailers (e.g., Mr Fresh and 7 Fresh).

#### 3.1.2. Determination of Discrete Choice Tasks

The DCE design for this study was generated using Ngene software (v1.2). A total of 144 choice scenarios were generated, representing the combination of the attributes. The primary effects of the attribute were estimated through a fractional factorial D-optimal design. The D-efficiency of 95.53% achieved demonstrates the quality of the experimental design [42,43]. Following the recommendation of Adamowicz et al. [44], a “Neither product” option was incorporated in the DCE to more accurately reflect real-world decision-making situations. The addition of a dummy-coded alternative specific constant (ASC) is a key feature of the DCE, as it enhances the realism of the choice task by considering situations where not all potential options are available [45]. With the inclusion of the ASC, the DCE produced a total of 72 choice set scenarios, divided into 9 groups (each with 8 tasks), to reduce respondent fatigue and cognitive burden. Each choice scenario presents two brisket options with different attribute levels and a “Neither product” (opt-out) option. Table 2 gives a sample choice set from the DCE.

### 3.2. Data Collection

Data for the DCE were collected through structured surveys between October and November 2019 in Beijing, Shanghai, Guangzhou and Shenzhen—four metropolitan cities in China. These four cities were selected for their economic scale and high internet penetration rates. The surveys were conducted in large shopping malls that housed supermarkets or offline grocery stores operated by OC retailers selling Australian beef products. Onsite data collection was conducted using paper-based questionnaires with predefined choice sets. Trained enumerators randomly intercepted consumers either at the entrances or within supermarkets and offline OC stores (e.g., Mr Fresh and 7 Fresh). Enumerators explained the choice experiment to ensure that participating consumers understood the attributes and associated levels. The choice experiment simulated real shopping scenarios without real money or actual products. A cheap-talk strategy (i.e., direct and costless communication) was adopted [46] to engage with respondents to interpret the attributes and their sub-levels. Participants were randomly given one of nine questionnaire versions, ensuring balanced distribution across locations. After excluding incomplete responses, the final dataset included 872 valid questionnaires spanning Beijing (232), Shanghai (221), Guangzhou (219) and Shenzhen (200), which is sufficient for statistical analysis [47].

### 3.3. Data Analysis

The data were first managed in Excel spreadsheets, then imported into Stata 15 and subsequently analysed using Nlogit 6.0. Model estimation began with a multinomial logit model, followed by a random parameter logit model using 1000 Halton draws for improved simulation. A latent class model was further employed to capture discrete preference heterogeneity. Lastly, consumers’ willingness to pay for different Australian beef (brisket) attributes was calculated.

#### 3.3.1. Multinomial Logit Model

The multinomial logit (MNL) model assumes that the random error term *ϵ*_*ni*_ follows an extreme value Type I (Gumbel) distribution, with independent and identically distributed (IID) errors distributed across alternatives [48]. The MNL model assumes IID error terms following a Gumbel distribution [40]. The probability of consumer n selecting alternative i is depicted as:

(5) $$P_{ni}=\frac{exp(\beta V_{ni})}{\sum_{j=1}^{J}exp(\beta V_{ni})}$$

for *i* = 1, …, *J*. When consumer n chooses i, the likelihood function of the LC model is:

(6) $$L=\prod_{n=1}^{N}\prod_{i=1}^{J}{\left(\frac{exp(\beta V_{ni})}{\sum_{j=1}^{J}exp(\beta V_{ni})}\right)}^{y_{ni}}$$

If consumer n chooses the alternative i, *y*_*ni*_ = 1; otherwise, *y*_*ni*_ = 0.

#### 3.3.2. Random Parameter Logit Model

The random parameter logit (RPL) model captures the heterogeneity in preferences by allowing parameters to differ across individuals [49]. The *V*_*ni*_ is:

*V_ni_* = 𝛽^′^*X_ni_* (7)

where the *β*′ is a vector of the random parameter capturing individual-specific preference for attributes. *X*_*ni*_ denotes the attribute vector associated with alternative i. As per Train [50], the likelihood of consumer n selecting alternative i can be described as:

(8) $$P_{ni}=\int \frac{exp(\beta V_{ni})}{\sum_{j=1}^{J}exp(\beta V_{ni})}f(\beta )d\beta \;$$

The product-specific parameters were assumed to follow a normal distribution and were dummy coded. The “Neither product” (opt-out) option was considered a fixed parameter to enhance model accuracy [47]. The price attribute was treated as a constant based on the approach of Hensher et al. [51]. Shopping channel 1 (the e-commerce marketplace), BRAND 1 (Chinese brand, manufactured in China) and traceability level 3 (no traceability) were defined as the reference levels for each attribute. To ensure that all estimated results of the RPL model yielded positive signs, BRAND 2 (Australian brand, manufactured in China) was specifically used as the reference level for the Beijing sample.

#### 3.3.3. Latent Class Model

The latent class (LC) model identifies consumer segments with homogeneous preferences within each class. It uncovers any underlying patterns of consumer preferences within and across these latent classes [52]. In the LC model, *f*(*β*) is discrete and takes on S distinct values. The probability of consumer n selecting alternative i is:

(9) $$P_{ni}=\sum_{s=1}^{S}\frac{exp\;\left(\beta s^{\prime }X_{ni}\right)}{\sum_{j=1}^{J}exp(\beta s^{\prime }X_{nj})}Rns$$

where *βs*′ represents the vector of parameters for class s, and *Rns* is the likelihood of consumer n belonging to class s. The probability is formulated as:

(10) $$R_{ns}=\frac{exp\;(\theta s^{\prime }Zn)}{\sum_{r}exp(\theta s^{\prime }\;Zn)}$$

where *θs* denotes the vector of parameters for consumers in class s, while *Zn* refers to the set of characteristics that influence consumer n’s membership in a particular class.

#### 3.3.4. Willingness to Pay

The willingness to pay (WTP) for choice attributes is calculated as:

(11) $$WTP=-\frac{\beta_{k}}{\beta_{p}}$$

where *β*_*k*_ is the coefficient of the attribute k, while *β*_*p*_ is the coefficient of the price attribute [42,43].

## 4. Results

### 4.1. Socio-Demographic Analysis

The DCE survey respondents were dominated by females, reflecting the cultural practice in China, where women often take responsibility for shopping and cooking. Most respondents were aged 26–30 and 30–40. More than 70% of respondents had a bachelor’s degree or above, indicating that most respondents had the necessary knowledge to understand the hypothetical options given in the DCE scenarios [47]. The largest group of respondents had a monthly taxable income ranging from CNY 6000 to 10,000. Married respondents accounted for 50–60% of the sample population. Most respondents indicated having 3–4 family members, and about half reported having no children. Table 3 depicts the socio-demographics of the survey respondents. While these socio-demographic profiles were not integrated with the latent class segments for more detailed analysis, their presentation shows the richness of our sampling.

### 4.2. Discrete Choice Selection: The Results of MNL Model and RPL Model

The model estimation shows that the RPL model performs better than the MNL model. Therefore, Table 4 reports the results of the RPL models across four first-tier cities of China.

#### 4.2.1. Shopping Channel Choice

Across all four cities, the coefficients for self-operated e-commerce stores (CHAN 1), OC mobile apps (CHAN 3) and OC offline grocery stores (CHAN 4) are all positive, ranging from 0.03572 to 1.70540. In Beijing and Guangzhou, CHAN 4 is significant, with coefficients of 0.97407 and 1.70540, respectively. In Shanghai, both CHAN 3 and CHAN 4 are significant at 0.30980 and 0.54265. However, in Shenzhen, none of the coefficients are significant, likely due to advanced infrastructure and shopping convenience across channels, reducing differences. These findings indicate consumer preferences for OC options in Beijing, Shanghai and Guangzhou, with a stronger inclination towards offline grocery stores (CHAN 4) compared to apps (CHAN 3). Within traditional e-commerce, consumers prefer self-operated stores (CHAN 1) over third-party ones.

#### 4.2.2. Brand and Manufacturer Location

The Australian-sourced brisket manufactured in Australia under an Australian brand (BRAND 3) exhibits a significantly positive preference among consumers across all four cities, with the coefficient values ranging from 0.30901 to 0.77913. This reveals that Chinese consumers in metropolitan cities have a significantly stronger preference for BRAND 3 compared to BRAND 2 (Australian-brand, manufactured in China) or BRAND 1 (Chinese-brand, manufactured in China), though Australian-sourced brisket applied to all brands. This underscores that brand and manufacturer location is an important product quality attribute to Chinese consumers, and the Australian-sourced brisket with the Australian brand, manufactured in Australia, is most valued by Chinese consumers compared to other alternatives.

#### 4.2.3. Country-of-Origin Traceability

The coefficients for the QR code and MLA country-of-origin icon are consistently positive and statistically significant across all four cities, ranging from 0.50310 to 1.97351. This suggests that Chinese consumers across all four cities have a favourable preference for the inclusion of communication of country-of-origin traceability. It should be particularly noted that the coefficient for the MLA’s “True Aussie” label is higher than that of the QR code. This implies that Chinese consumers tend to prefer and trust the MLA origin icon more than the QR code approach.

### 4.3. Different Consumer Clusters: The Results of Latent Class (LC) Model

Preference variations among consumers for Australian brisket in China, as illustrated by the RPL model, were further analysed to identify different consumer clusters. Table 5 shows the distribution of the consumer cluster across four latent classes (clusters).

The first latent cluster (23%) values premium product quality attributes, with significant coefficients for OC offline stores, Australian-sourced brisket segmented and packaged in Australia and the MLA traceability label while showing a positive price coefficient. These “premium buyers” prioritise high-quality features over price, aligning with the belief that good products are not cheap. The second cluster focuses on shopping channels and traceability, with higher utility derived from these attributes and a smaller yet significant coefficient for Australian-manufactured and branded brisket. These “channel and traceability-conscious” buyers prioritise convenience and transparency in their purchases. The third cluster (26%) prefers OC mobile apps and offline stores but is also highly price-conscious. These “OC and price-conscious” consumers balance shopping convenience with affordability. The fourth cluster (30%) strongly prefers the OC mobile app, showing high coefficients for Australian-manufactured and branded brisket and traceability. These “tech-savvy and discerning” shoppers favour traceable premium brisket and rely on mobile apps for their purchases, emphasising product transparency and traceability due to the lack of direct interaction in their shopping experiences.

### 4.4. Willingness to Pay (WTP) for Different Attributes

**Figure 1:**
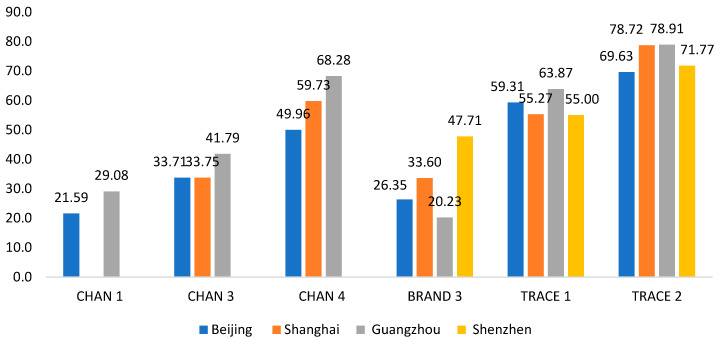
Willingness to pay for different brisket product attributes (unit: CNY/500 g). Note: CNY 1 ≈ AUD 0.206 ≈ USD 0.14 at the time of the study. CHAN 1: Self-operated e-commerce stores; CHAN 3: OC mobile apps; CHAN 4: OC offline grocery stores; BRAND 3: Australian brand, manufactured in Australia; TRACE 1: QR code; TRACE 2: MLA “True Aussie Beef” certification.

Figure 1 illustrates WTP for different attribute levels of Australian brisket products.

Chinese consumers’ WTP for different shopping channel attributes is basically significant, except for the coefficients of Shenzhen and Channel 1 in Shanghai. Among the selected shopping channels, CHAN 4 has the highest WTP, with values of CNY 49.96, CNY 59.73 and CNY 68.28 for Beijing, Shanghai and Guangzhou, respectively, averaging CNY 59.32 (approx. AUD 12.22). CHAN 3 ranks second, with WTP values of CNY 33.71, CNY 33.75 and CNY 41.79 for Beijing, Shanghai and Guangzhou, respectively, averaging CNY 36.42 (AUD 7.50). The WTP for CHAN 1 is CNY 21.59 for Beijing and CNY 29.08 for Guangzhou, averaging CNY 25.33 (AUD 5.22).

WTP for BRAND 3 (Australian brand, manufactured in Australia) was calculated only as the coefficients of attribute BRAND 2 and is negative in Beijing, Shanghai and Guangzhou. In Shenzhen, it is significant but lower than the coefficient of BRAND 3. The WTP for BRAND 3 ranged from CNY 20.23 to CNY 47.71 in four cities, and the average WTP for this attribute level is CNY 31.97 (AUD 6.59).

Country-of-origin traceability is crucial for Chinese consumers’ appreciation of Australian beef. The MLA “True Aussie Beef” label (TRACE 2) has the highest WTP, ranging from CNY 69.63 in Beijing to CNY 78.91 in Guangzhou, with an average of CNY 74.76 (approx. AUD 15.40) across the four cities. The QR code (TRACE 1) ranks second at CNY 58.37 (AUD 12.02), highlighting consumers’ demand for origin traceability. Consumers prefer the MLA logo over detailed QR codes, valuing the assurance of origin, with “Australia-made” itself driving willingness to pay a premium.

## 5. Discussion

### 5.1. Discussion of Results

This study used a discrete choice experiment (DCE) to address a gap in the literature around how omnichannel (OC) retailing interacts with product quality attributes in influencing consumer behaviour within the context of Australian beef (brisket) purchases in China. This study distinguishes itself from previous studies [53,54,55,56] by offering new insights into the dynamics of consumer preferences and e-retail strategies, especially for emerging OC retailing. The emergence of OC retailers in China, such as Mr. Fresh and 7-Fresh, further underscores the timeliness and relevance of this research. Another novel aspect of this study lies in its dual examination of channel and product quality attribute preferences in e-retail. Additionally, the study explores the impact of two traceability options—MLA’s “True Aussie Beef” logo and QR codes—on consumer preferences and willingness to pay (WTP), addressing gaps in understanding how Chinese consumers perceive traceability labels in the imported beef sector within the food e-retail landscape.

This study also contributes to the existing knowledge of food OC and consumer-oriented marketing in the e-retail environment by revealing that Chinese consumers place significant value on selected attributes, including shopping channels, brand and manufacturer location, and origin traceability. Beyond aligning with Zisper et al. [27] in revealing Chinese consumers’ preference for OC retailing in Australian beef (brisket) purchases, this study found that the brand, manufacturer location, and origin traceability emerged as critical attributes in food e-retail. Chinese consumers’ preference for Australian-branded products, segmented and packaged in Australia, reflects the importance of brand origin as a quality signal [11,12]. Chinese consumers’ willingness to pay a premium for traceability information is consistent with prior studies [55,57,58] to reflect its importance in enhancing perceived product quality. The results also align with contemporary studies on the interplay between country of origin, pricing strategies and brand reputation [17,18].

Through the latent class analysis, this study identified four distinct consumer segments: (i) premium shoppers, (ii) channel and traceability-oriented shoppers, (iii) OC and price-oriented shoppers, and (iv) tech-savvy and discerning shoppers. Notably, premium shoppers displayed positive price sensitivity, indicating a willingness to pay more for high-quality attributes. In contrast, the other three segments exhibited price-conscious behaviour. These segmented consumer profiles provide actionable insights for tailoring food marketing strategies, particularly in aligning OC offerings with traceability and brand communication to different consumer preferences. The WTP analysis further demonstrates a robust preference for OC retailing, with consumers paying approx. 44% and 134% more per 500 g, respectively, compared to self-operated e-commerce stores (CNY 25.33 ≈ AUD 5.22). The higher WTP for OC offline stores is likely due to enhanced shopping experiences, as suggested by Chopra [5]. The preference for OC shopping also underscores the increasing sophistication of Chinese consumers in integrating online convenience with offline assurance, a trend that parallels findings by Wang et al. [8].

### 5.2. Strategic Contributions to International Food E-Retail

This study makes significant strategic contributions to the international food e-retail and marketing setting, particularly in understanding how OC retailing and product quality attribute signals interact to shape consumer behaviour, including purchase preferences and WTP within the context of Australian beef purchases in China.

First, it advances the understanding of Chinese consumers’ e-retail channel preferences and how OC retailing influences consumer preferences and WTP. By examining consumer channel preference in various e-retail settings through the lens of Australian brisket purchases in China, this study found that Chinese consumers prefer purchasing Australian brisket via OC mobile apps and offline stores and are willing to pay premiums than in conventional e-commerce. This underscores the strategic importance of embracing OC in food retailing in China, where e-retail is increasingly central to everyday shopping.

Second, this study contributes to the attribute signalling literature by revealing that product attributes like the MLA “True Aussie Beef” logo, traceable QR codes, and the Australian origin of products significantly impact consumer choices and justify premium pricing. It also highlights the strategic importance of effectively communicating key product quality attributes—such as brand and manufacturer location and traceability information—to influence consumer decision-making in the food e-retail setting. These insights extend prior studies by integrating these product quality attributes as a critical value proposition in retail. It provides a framework to align attribute communication with e-retail channels, including OC, which is strategically important for value creation and customer services.

Finally, this study extends international food marketing research by identifying four distinct consumer segments: (i) premium shoppers, (ii) channel and traceability-oriented shoppers, (iii) OC and price-oriented shoppers, and (iv) tech-savvy and discerning shoppers. These segments reveal differentiated consumer responses to OC shopping and product quality attribute signals, offering a nuanced framework for food marketers to tailor e-retail strategies in Chinese contexts. These insights support Australian beef producers and marketers in optimising their operational strategies, enhancing their market competitiveness, and ensuring sustainable growth in China’s evolving e-retail environment.

### 5.3. Practical Implications for International Food E-Retail

This study offers several practical insights into how Australian beef producers can enhance their competitiveness in the Chinese market through OC retailing. The socio-demographic analysis of the sample population provides critical strategic insights into consumer segments, enabling businesses to design communication strategies and promote the right products to the right consumers [59,60]. By investigating consumer preferences and WTP for Australian brisket products based on various attributes in the food e-retail context, this study shows the value of integrating shopping channels with branding, manufacturer location and traceability information.

Another implication is that Australian beef producers and marketers should consider adapting their operations to fit the Chinese retail landscape by, for example, adding production lines to cater specifically to Chinese e-retail shoppers. Products on e-commerce platforms often feature customised packaging rather than bulk or quarter cuts, aligning with the demand for segmented, ready-to-use products packaged directly in Australia. Adapting to these preferences can enhance the market competitiveness of Australian beef and meet consumer expectations for convenience and quality. Furthermore, our findings reveal that product quality attributes, such as brand and manufacturer location and country-of-origin labels, significantly influence consumer preferences and justify premium pricing in retail. These insights inform businesses about developing targeted marketing communication approaches to resonate with consumer priorities.

This study also offers valuable guidance for improving customer service and creating additional value for stakeholders within the Australian beef supply chain. The preferences identified in this study for Australian beef via OC retailing can also provide broader insights into the marketing and distribution of other foods and agricultural products. Additionally, these findings may offer valuable insights into products imported from countries other than Australia, providing a global perspective on OC retailing and consumer behaviours. The insights on Chinese consumer behaviours present an opportunity for Meat & Livestock Australia (MLA) and Australian beef producers to solidify their market position. The Australian beef industry can strengthen its competitive edge by aligning with Chinese OC retailing practices and emphasising consumer-valued quality attributes. This alignment offers the potential to influence marketing practices in other international markets and industries.

### 5.4. Limitations and Future Research

This study has several limitations that should be noted. First, the study was conducted in late 2019 before China’s ban on Australian beef imports and the global pandemic. Although underlying consumer preferences for brand, manufacturer location, traceability and OC access remain highly relevant, significant changes in China’s online retail environment, consumer behaviours and OC strategies and practices may have occurred, particularly influenced by import bans and COVID-19. With the import ban lifted in 2024, future research is suggested to build upon this study to understand dynamic changes in e-retail and inform optimal re-entry strategies in these post-pandemic times.

Second, this study primarily defined OC retailing as a combination of mobile apps and physical grocery stores, which may oversimplify the diverse and integrated nature of modern OC strategies in China. Future studies could expand the definition of OC channels to include a wider array of touchpoints and explore varying levels of integration to better represent the current landscape.

Third, the study’s geographic scope means the results may not be generalisable to the broader Chinese market. While these four first-tier cities (Beijing, Shanghai, Guangzhou and Shenzhen) are crucial to the market, consumer preferences and behaviours can vary significantly in lower-tier cities. Future research could incorporate data from a wider range of cities, including lower tiers, to provide regional differences in willingness to pay and attribute sensitivities.

Fourth, this study focused on available extrinsic product cues in e-retail environments without conducting direct sensory testing of intrinsic product attributes. Future research could incorporate in-store experiential or guided sensory trials to complement extrinsic cue analysis in e-retail consumer choices.

Another limitation is the lack of understanding of how differences in income, lifestyle and education across city tiers influence consumer preferences. Future studies could enrich the latent class analysis by modelling the socio-demographic predictors of consumer segments to better understand the heterogeneity across different consumer groups.

Additionally, this study focused specifically on Australian beef (brisket in particular), a product that is important in the market but may not fully represent consumer preferences for other meat cuts or food categories. Repeating this study with other products, such as different cuts of beef or other types of food products, could help assess the robustness of the findings across various food categories and retail environments.

Finally, this study used a DCE based on hypothetical purchase scenarios. However, it might not fully reflect actual consumer behaviours. The potential discrepancy between stated preferences in hypothetical scenarios and real-world purchase decisions should be considered. To validate the findings, future research could combine DCE results with actual purchase data or conduct behavioural experiments that involve real transactions.

## 6. Conclusions

This study examined how OC retailing interacts with product quality attributes, including shopping channels, brands and manufacturer locations and country-of-origin traceability labels to influence Chinese consumers’ preferences and willingness to pay (WTP) for Australian beef (brisket) within the food e-retail context. The study found that Chinese consumers had a higher preference for OC mobile apps and offline stores. Origin traceability label, particularly the MLA “True Aussie Beef” icon, was the most significant quality attribute that Chinese consumers were willing to pay extra for. Furthermore, Australian-sourced beef, carrying an Australian brand and manufactured in Australia, was also a significant attribute that Chinese consumers preferred. Additionally, the study identified four latent classes of consumers with different preferences for price, shopping channels, brands and manufacturer locations and traceability, which can be respectively categorised as premium consumers, channel and traceability-oriented consumers, and price-oriented consumers, as well as tech-savvy and discerning consumers.

This study provides valuable insights for enhancing international marketing strategies and improving the competitiveness of Australian beef products in the Chinese market. With the growing significance of OC retailing in consumer purchasing behaviours generally [61], adopting an OC strategy is becoming essential in selling Australian beef products in China. Alongside channel selection, the study has indicated that other product attributes, such as brand and manufacturer location and country-of-origin traceability information, hold great importance for Chinese consumers and could influence consumers’ WTP. The study further offers novel evidence on how omnichannel retail and extrinsic product quality attributes drive consumer preferences and WTP for Australian beef in China and highlights the strategic importance of traceability labels, OC platform integration and branding communication in shaping consumer value perception. These findings have significant implications for MLA and the Australian beef industry, suggesting the need to align with the Chinese OC retailing and focus on the desired product attributes in OC communication. Furthermore, the identification of four discrete consumer segments provides actionable guidance for segment-specific strategies. The emerging integrated shopping trends, together with consumer segmentation in China’s e-retail landscape, present an opportunity for beef and other food exporters and Chinese OC pioneers, potentially influencing other international markets and industries.

## Figures and Tables

**Table 1:** Attributes and corresponding levels for 500 g Australian beef brisket.

Attribute	Number of Levels	Specific Attribute Level					
Shopping Channel [32]	4	(1) Traditional e-commerce: self-operated e-commerce store (2) Traditional e-commerce: 3rd-party store on an e-commerce platform (3) New omnichannel (OC): Purchase via mobile app (4) New omnichannel (OC): Purchase at a physical grocery					
Brand and Manufacturer Location [39]	3	(1) Australian brand, manufactured in Australia (2) Australian brand, manufactured in China (3) Chinese brand, manufactured in China					
Country-of-Origin Traceability [19]	3	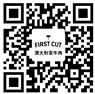 QR code-enabled traceability		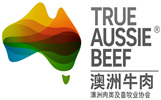 MLA origin label			None
Price [10]	4	CNY 35	CNY 50		CNY 65	CNY 80	

Note: All brand options are Australian-sourced beef; CNY 1 ≈ AUD 0.206 ≈ USD 0.14 at the time of the study.

**Table 2:** A sample choice set for brisket.

Attribute	Brisket Choice 1	Brisket Choice 2	Brisket Choice 3
Shopping Channel	Purchase from e-commerce marketplace	Purchase from new omnichannel (OC) offline stores	None
Brand and Manufacturer Location	Australian brand, manufactured in China	Australian brand, manufactured in China	
Country-of-Origin Traceability	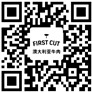 QR code-enabled traceability	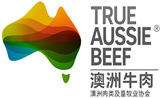 MLA origin label	
Price (per 500 g)	CNY 50	CNY 80	
I would buy:	o	O	o

Note: All brand options are Australian-sourced beef; CNY 1 ≈ AUD 0.206 ≈ USD 0.14 at the time of the study.

**Table 3:** Socio-demographic profiles of the sample.

Profile	Beijing	Shanghai	Guangzhou	Shenzhen	Overall
Number of respondents	232	221	219	200	872
Gender distribution—Female (%)	59.1	53.8	68	56.5	59.4
Age group breakdown (%)					
18–25	14.2	17.6	29.2	12.5	18.5
26–30	21.6	19.5	28.3	29	24.4
31–40	37.5	20.4	31.1	44	33
41–50	17.7	16.7	9.1	13	14.2
51–60	6.9	15.8	1.8	1.5	6.7
Above 60	2.2	10	0.5	0	3.2
Education level (%)					
Middle school and below	0.4	2.3	0.5	0	0.8
High school	13.4	26.7	3.7	3.5	12
Bachelor	60.3	53.4	68	73	63.4
Master	24.6	16.7	25.1	22	22.1
PhD and above	1.3	0.9	2.7	1.5	1.6
Taxable monthly income range (%)					
Below 3000	4.3	4.5	6.8	2	4.5
3000–5999	15.9	26.7	16	10.5	17.4
6000–9999	31.5	36.2	30.1	36.5	33.5
10,000–14,999	18.1	14	24.2	24	20
15,000–19,999	15.5	6.8	9.6	15.5	11.8
20,000–29,999	9.1	5.4	8.7	6.5	7.5
30,000–49,999	5.6	4.5	3.2	4	4.4
Above 50,000	0	1.8	1.4	1	1
Marital status—Married (%)	67.7	61.5	51.6	69.5	62.5
Family size breakdown (%)					
1–2	22	32.6	18.7	19	23.2
3–4	63.8	59.7	62.1	74	64.7
5 and above	14.2	7.7	19.2	7	12.2
Number of children (%)					
None	47.4	62.4	53.9	46	52.5
1	41.8	31.2	32.4	47	38
2	10.3	6.3	12.3	7	9.1
3 and above	0.4	0	1.4	0	0.5

Note: CNY 1 ≈ AUD 0.206 ≈ USD 0.14 at the time of the study.

**Table 4:** Parameter estimates from the RPL models by city.

Variable	Beijing (BJ)	Shanghai (SH)	Guangzhou (GZ)	Shenzhen (SZ)
CHAN 1	0.42561 *** (0.12966)	0.05481 (0.10144)	0.72553 *** (0.14073)	0.03572 (0.11458)
CHAN 3	0.65565 *** (0.13288)	0.30980 *** (0.10896)	1.04417 *** (0.13515)	0.13188 (0.13760)
CHAN 4	0.97407 *** (0.14490)	0.54265 *** (0.11455)	1.70540 *** (0.16655)	0.10757 (0.12692)
BRAND 1 (BJ)	0.02374 (0.12010)	-	-	-
BRAND 2 (SH, GZ, SZ)	-	0.12393 (0.08920)	0.10527 (0.11524)	0.34181 *** (0.10887)
BRAND 3	0.50948 *** (0.12075)	0.30901 *** (0.09099)	0.50936 *** (0.13054)	0.77913 *** (0.11691)
TRACE 1	1.14493 *** (0.12492)	0.50310 *** (0.08218)	1.58314 *** (0.14004)	0.89612 *** (0.10508)
TRACE 2	1.36139 *** (0.12880)	0.71590 *** (0.09025)	1.97351 *** (0.15298)	1.17085 *** (0.11343)
Non-random parameters in utility functions				
PRICE	−0.01949 *** (0.00284)	−0.00906 *** (0.00232)	−0.02500 *** (0.00314)	−0.01639 *** (0.00275)
NONE	−1.12899 *** (0.19990)	−2.19088 *** (0.19309)	−0.70530 *** (0.20480)	−1.18719 *** (0.19745)
Standard deviation of the random parameters				
CHAN 1	0.61783 *** (0.21754)	0.36360 (0.23404)	0.55911 ** (0.26618)	0.00450 (0.40929)
CHAN 3	0.72870 *** (0.19952)	0.63058 *** (0.18173)	0.17674 (0.52786)	0.99348 *** (0.17812)
CHAN 4	0.80956 *** (0.19132)	0.32119 (0.28030)	0.75154 *** (0.20355)	0.14476 (0.43376)
BRAND 2	0.98281 *** (0.15813)	0.56915 *** (0.13508)	0.63862 *** (0.18066)	0.70299 *** (0.14227)
BRAND 3	0.97395 *** (0.15201)	0.60096 *** (0.14570)	1.06528 *** (0.15400)	0.86175 *** (0.13603)
TRACE 1	0.96872 *** (0.15753)	0.24525 (0.22843)	0.93989 *** (0.15275)	0.51082 *** (0.15450)
TRACE 2	0.96537 *** (0.15056)	0.31019 (0.19561)	0.94789 *** (0.15327)	0.60704 *** (0.15184)
Summary statistics				
Observations	1856	1768	1752	1600
McFadden pseudo R2	0.2413985	0.2814263	0.3075762	0.2138844
Log likelihood	−1546.80691	−1395.71917	−1332.75575	−1381.81805
Inf. Cr. AIC	3125.6	2823.4	2697.5	2795.6

Note: *** and ** indicate significance at 1% and 5% levels, respectively. Numbers in parentheses are standard errors. CHAN 1: Self-operated e-commerce stores; CHAN 3: OC mobile apps; CHAN 4: OC offline grocery stores; BRAND 1: Chinese brand, manufactured in China; BRAND 2: Australian brand, manufactured in China; BRAND 3: Australian brand, manufactured in Australia; TRACE 1: QR code; TRACE 2: MLA origin certification; NONE: Neither product.

**Table 5:** Parameter estimates from the LC model across the four cities.

Variable	Cluster 1	Cluster 2	Cluster 3	Cluster 4
CHAN 1	−0.00764 (0.13108)	0.86888 *** (0.15662)	0.11760 (0.14355)	0.27033 * (0.16086)
CHAN 3	0.10346 (0.12638)	1.06062 *** (0.15985)	0.48819 *** (0.12763)	0.62451 *** (0.17241)
CHAN 4	0.35581 ** (0.14601)	1.59039 *** (0.16367)	0.97312 *** (0.16827)	0.45103 ** (0.20346)
BRAND 2	0.04658 (0.09802)	0.00532 (0.11452)	0.16086 (0.12239)	0.47132 *** (0.12861)
BRAND 3	0.33936 *** (0.12013)	0.33494 *** (0.12360)	0.33060 ** (0.12905)	1.12470 *** (0.16140)
TRACE 1	0.15007 (0.12234)	1.27166 *** (0.14816)	0.16844 (0.12599)	2.39117 *** (0.21848)
TRACE 2	0.35975 *** (0.12082)	1.35688 *** (0.15269)	0.37536 *** (0.13626)	2.82650 *** (0.23407)
PRICE	0.03138 *** (0.00431)	−0.02593 *** (0.00344)	−0.06338 *** (0.00569)	−0.01628 *** (0.00513)
NONE	−1.26030 *** (0.42901)	0.65184 ** (0.26305)	−7.01139 *** (0.61615)	−0.68787 * (0.39605)
Summary Statistics				
Class Prob.	0.22866 ***	0.20719 ***	0.26076 ***	0.30339 ***
McFadden Pseudo R2	0.3099125			
Log Likelihood	−5288.77461			
Inf. Cr. AIC	10,655.5			

Note: ***, ** and * denote significance at the 1%, 5% and 10% levels, respectively. Numbers in parentheses represent standard errors. Cluster 1: Premium shopper; Cluster 2: Channel and traceability-oriented shopper; Cluster 3: Omnichannel (OC) and price-oriented shopper; Cluster 4: Tech-savvy and discerning shopper.

## Data Availability

The original contributions presented in the study are included in the article; further inquiries can be directed to the corresponding authors.

## Abbreviations

The following abbreviations are used in this manuscript:

ASC	Alternative specific constant
DCE	Discrete choice experiment
IID	Independent and identically distributed
LC	Latent class
MLA	Meat & Livestock Australia
MNL	Multinomial logit
OC	Omnichannel
RPL	Random parameter logit
WTP	Willingness to pay
